# Premature alarm on the impacts of climate change on Arctic Char in Lake Hazen

**DOI:** 10.1038/s41467-018-06479-5

**Published:** 2018-09-28

**Authors:** Jean-Sébastien Moore, Jacqueline M. Chapman, Marc J. Mazerolle, Les N. Harris, Eric B. Taylor

**Affiliations:** 1 Département de biologie, Institut de biologie intégrative et des systèmes, Centre d’études nordiques, Ressources aquatiques québec, Université Laval, Québec, Canada; 2 Department of Biology, Fish Ecology and Conservation Physiology Laboratory, Carleton University, Ottawa, Canada; 3 Centre d’étude de la forêt, Département des sciences du bois et de la forêt, Université Laval, Québec, Canada; 4 Fisheries and Oceans Canada, Central and Arctic Region, Winnipeg, Canada; 5 Department of Zoology, Biodiversity Research Centre, and Beaty Biodiversity Museum, University of British Columbia, Vancouver, British Columbia Canada

A recent paper by Lehnherr et al.^1^ reported on a long-term study of the ecological impacts of climate change in the world’s largest high Arctic lake: Lake Hazen on Canada’s Ellesmere Island. The paper made a convincing case that climate change has had a dramatic and significant impact on the watershed of this important freshwater ecosystem. Some of these changes have clearly impacted the ecology of Lake Hazen. We disagree, however, with the conclusion that such ecological changes have resulted in a significant decline in the condition of Arctic Char (*Salvelinus alpinus*) from the lake based on the presented data. It is critical to examine the evidence for changes in the condition of Arctic Char given the importance of this species to communities throughout Canada’s Arctic as a valued food resource and because changes to condition could impact its management.

**Fig. 1:**
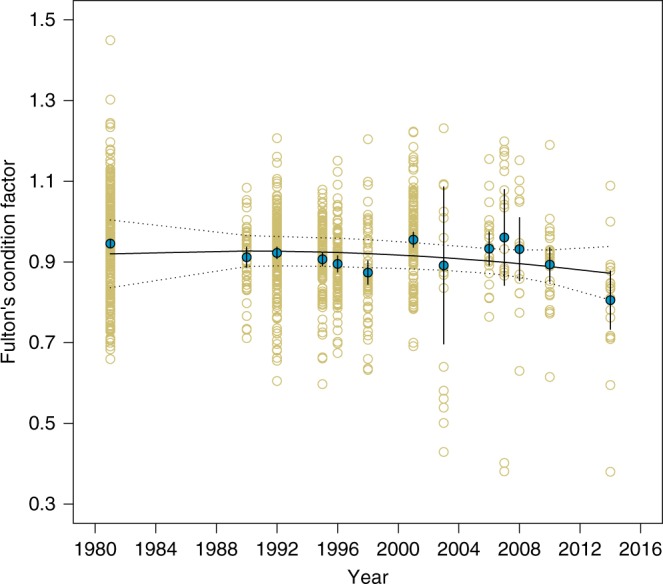
Fulton’s condition factor of Arctic Char collected between 1981 and 2014 from Lake Hazen. Open circles correspond to the condition factor of individuals, whereas solid blue circles are annual averages ± 1 SE. The solid line shows predictions from a quadratic regression of annual averages with 95% confidence intervals (*p* = 0.387)

Evidence for the impact of climate change on Arctic Char was presented in the form of a time-series (1981–2014; *N* = 13) of Fulton’s condition factor (Fig. 1), a widely used index of the wellbeing or robustness (i.e., mass relative to length) of individuals or populations of fish^2^ (for criticisms of the use of condition indices see refs. ^3,4^). The authors concluded that there was a significant decline in the condition factor of Arctic Char during these years as a result of climate-mediated changes in this ecosystem. We argue, however, that the statistical analysis used to assess the significance of that trend is problematic. We re-analyzed the presented data, which was graciously made available by the authors, using the same analysis as described in the paper. Here, we highlight three issues with the authors’ approach. First, the authors reported a significant quadratic regression model (*F*_2, 1133_ = 8.47, *p* = 0.0002 in our analysis), whereas the quadratic term itself was not significant (type 3 *F*_1, 1133_ = 0.1659, *p* = 0.684). Second, and more importantly, each individual measurement was treated by Lehnherr et al.^1^ as an independent data point in the analysis, artificially inflating the reported significance (a problem further accentuated by the unequal sample sizes among years). In the current data set, observations from the same year are likely to be more similar than observations from different years, violating the assumption of independent errors of multiple regression. This aspect of the data should have been treated with an alternative approach such as including a random effect of year or using the annual mean condition factor to test the hypothesis of a decline across years. For instance, the quadratic regression model working on the annual mean condition index is no longer significant (adjusted *R*^2^ = 0.0075; *F*_1, 10_ = 1.045; *p* = 0.387) and neither is a linear regression (adj. *R*^2^ = 0.047; *F*_1, 11_ = 1.597; *p* = 0.233).

Our final concern regards the very weak effect size of year. Specifically, there is a weak variation of the mean of Fulton’s condition factor across years (Fig. 1). Even after removing the quadratic term, the slope of the linear regression conducted on either the annual means of condition factor or on the individual fish is −0.002. Such a modest decline across years suggests that this change is not biologically significant, potentially due to greater within-year variation in the condition factor than among years. In fact, when fitting a linear mixed model to the entire data set (*N* = 1136) and treating the year as a random effect, we observe higher within-year variability (*σ*_residual_ = 0.116, 95% CI: [0.111, 0.121]) than the variability among years (*σ*_year_ = 0.032, 95% CI: [0.018, 0.057]). Therefore, even if statistical significance had been observed (which we contend it was not), such a weak effect of year is likely an example of a statistically significant result where the effect size is not biologically significant^5, 6^.

There are valid reasons to be concerned about the fate of Arctic Char in a rapidly changing Arctic^7–9^. The few long-term datasets available from the Canadian Arctic, however, do not suggest declines in abundance or body condition linked to climate change (e.g., refs. ^10, 11^). In fact, some studies even suggest a positive effect of an increased summer ice-free period on the condition of anadromous stocks^12^ (note however that the individuals studied in Lake Hazen are not anadromous). We conclude that the available evidence does not currently support the conclusion of a decline in condition factor of Arctic Char in Lake Hazen. Consequently, sounding the alarm on impacts related to climate-change on Arctic Char in northern Canada is premature. Continued monitoring of Arctic Char coupled with appropriate sampling designs and statistical analyses is, however, central to the effective management of this vital resource.
